# Radcliffe Infirmary, Oxford

**Published:** 1891-04-11

**Authors:** 


### The Radcliffe Infirmary, Oxford.

(By Our Special Commissioner.)

It is greatly to the credit of Englishmen that during the last quarter of a century, in every part of the country, an active interest has been displayed in the well-being of all the medical charities to be found in our large towns. In some it may truly be said that it is considered more of an honour and privilege to be connected with the management of the great medical institutions than to take part in the local government of the community. This feeling is, no doubt, largely due to the fact that sickness and suffering excite the warmest feelings of humanity and teach lessons which none but the most callous can afford to neglect or ignore. It has been our duty and privilege during the last twenty years to inspect hospitals and kindred charities, not only in every part of Great Britain, but in many foreign countries. We went to Oxford with a feeling that a great University town, famed for its culture and intelligence, must almost necessarily present the acme of perfection in hospital administration. Unfortunately, the facts proved to be altogether otherwise, and we never remember any more depressing experience than that attending our inspection of the Radcliffe Infirmary.

### Forgotten and Asleep.

The Radcliffe Infirmary is entered by the basement. The entrance is situated in the oldest part of the buildings, which present an altogether neglected and miserable appearance. The fittings and appliances are for the most part of the oldest and worst description, and the age of most of them tends to discourage those who are responsible for the cleanliness and general appearance of the whole place. We venture to think and to say that it would be difficult to find a parallel to the present condition of many parts of the Radcliffe Infirmary amongst the voluntary institutions of this country to-day. The general aspect is depressing, and the whole place displays an absence of "go" and brightness difficult to realise without a personal inspection of the institution. It is quite evident that very little real interest is taken in the institution by the inhabitants of Oxford, and we can only hope that those who are interested in, and have a sound knowledge of the best forms of hospital administration, will make an early opportunity to visit the Radcliffe Infirmary, in order to see what was the condition of many of our institutions thirty years ago. No doubt many other medical institutions could be improved, but it is matter for congratulation to feel, with a precise knowledge of the facts, that few, if any, hospitals have sunk to the one-horse condition of the Radcliffe Infirmary. So far as the nursing staff and others who are responsible for the cleanliness of the wards are concerned, they, no doubt, have done their best, under adverse circumstances, to keep the place clean and in order. Unfortunately, however, the condition of the wards, so far as the walls, the sanitary fittings, baths, and other appliances are concerned, exhibits an almost total disregard not only for appearances, but also for sanitary requirements. Thus in the chief surgical ward, which was regarded as a type of better things twenty years ago, the old original Parian cement remains in its pristine condition, having never been painted from first to last. Many years ago it was proved that Parian cement was one of the most absorbent of materials, and we venture to think that at least half the thickness of the plaster on the walls is now impregnated with impurities of various kinds. The mere discolouration of the Parian cement is an evil to be regretted, and its extent is graphically illustrated by comparing it with the new cement on the ceilings and walls where the old Parian has fallen down. In the Jubilee wing some walls have not been painted, and the aspect of the staircase and entrance hall is in consequence altogether unworthy of a great county hospital. Here again the fittings are obsolete, and instead of Rufford's baths, one has the old uninviting painted baths, which never have been, and never can be, made suitable for hospital purposes. Everywhere, indeed, in the hospital, except in the kitchen, operation theatre, and possibly in the children's ward (where there is an excellent Sister), the same absence of care and regard for appearances is painfully manifest.

### Probable Causes of Neglect.

The cardinal fault in the administration of this institution is probably to be found in No. 21 of the Rules and Orders, which provides "that it shall not be lawful for the Committee of Management to proceed to any new work, the cost of which shall exceed £20." In other words, the Committee of Management is really not trusted by the Governors to manage the affairs of the institution, and in consequence it is fair to assume that its members do not take that active interest in the efficiency of the institution which is to be found elsewhere, where the Governors have had the wisdom to endow the committees with adequate powers, and to trust them absolutely. Again, there would appear to be an absence of visitors from outside. The general public, *i.e.*, the inhabitants of Oxford, seem to consider it no part of their duty or privilege to actively interest themselves in the internal arrangements and management of the Radcliffe Infirmary. Thus we were informed that in the children's ward during the last two years there have been very few visitors to encourage the workers in the ward or to sympathise with the poor sufferers in the cots. Indeed, unless we have formed a wrong impression from what we saw and heard, the inhabitants of Oxford have yet to be aroused to the fact that it is their duty, as it ought to be regarded as their privilege, to spend and be spent in promoting the best interests of all who seek relief within the wards of the local infirmary. It is surely a sad reflection on the culture of Oxford, and on its civilisation, that the children's ward should occupy so small a portion of the attention of the inhabitants, and that they should practically leave the paid staff without encouragement—nay, without even the little additional adjuncts which, so far as we have experienced, are to be found in every other children's ward in every other town throughout the United Kingdom. Surely there is a danger that the fate which attended the excessive culture of the Athenians will befall this university town unless the men and the women who reside there are aroused to a sense of their duty, and determine to fulfil it. How it has come to pass that all the best instincts of our common humanity should have been allowed to lie dormant in Oxford we cannot, of course, divine. Be the cause what it may, we are perfectly confident that the Radcliffe Infirmary is not the only sufferer by the present state of things, but that the great majority of the citizens must lack much which they would otherwise possess by the inertness and disregard they show to the care and cure of the sick and suffering members of their community.

### The Nursing Department.

The Matron, Sisters, and nurses are undoubtedly devoted women, doing their work in an unostentatious and self-denying way. We took some trouble to satisfy ourselves as to their qualifications and work, and we arrived at the conclusion that both were very good. The accommodation, however, provided for the nurses is altogether behind the times. The cubicles, even the best of them, which are at present occupied by the nurses leave much to be desired, especially as regards the sanitary arrangements and ventilation. Several of these cubicles ought to be condemned, as they do not possess a sufficiency of air or light. The two baths devoted to the nurses are in a condition so untempting as to be disgraceful, and we would advise the authorities to condemn them and substitute for them Rufford's porcelain baths without delay. The sitting-rooms given up to the staff might be much more comfortably furnished, and the nurses' library requires re-stocking and re-arrangement. All these things are the more remarkable because it appears from the report that during the year 1890 the institution received no less a sum than £458 from premiums paid by probationer nurses, and a further sum of £151 earned by the nursing staff. We would point out that the day has gone by when the public will permit an institution to derive profit from its nurses without giving in return adequate accommodation and a sufficient provision by way of pensions and sick-pay to secure that no nurse employed by it shall be in want in the day of sickness or old age.

### Much-needed Improvements.

To bring the Radcliffe Infirmary up to a level with other county institutions in this country the following alterations are imperatively called for: The front block should be entirely reconstructed or built from the foundation, and new fittings should be supplied to every portion of the building—ward sinks, baths, and other appliances all needing renewal. The wards situated in the out-lying blocks, if the fittings are modernised, may be left as they are, provided an adequate regard is shown for internal appearances in the way of repainting and renewal. A properly-constructed nurses' home is a *sine quâ non*, and until this is forthcoming those responsible for the administration of the Radcliffe Infirmary cannot be held to adequately realise their responsibilities or to discharge them. More personal interest on the part of the inhabitants of Oxford in all that concerns the internal arrangement and management of the Infirmary must be excited. It is perfectly evident that there is a lamentable absence of individual energy and continuous effort to maintain this institution in a high state of efficiency. These things ought not to be. Every community owes a debt of gratitude to its hospital, because there the members of the medical profession who minister to the needs and sufferings of the well-to-do portion of the community learn much of their business, and by its existence they are enabled to maintain a high degree of efficiency and knowledge, without which the best interests of the population must suffer.

### To the Intelligent Resident.

We have written this account of a visit to one of our oldest county infirmaries from a strong sense of public duty, and we can honestly say that in the course of an experience extending over a quarter of a century we never remember to have visited a hospital with a feeling of greater interest or to have left it with a feeling of greater depression and astonishment. We cannot believe that any intelligent resident in Oxford who will pay a visit to the Infirmary with an open mind, and who makes his inspection in the light of what we have here stated, will rest content until all the suggested alterations have been effectually carried out. Until such a change takes place the Radcliffe Infirmary must be regarded as an obsolete institution, remarkable from the fact that it has been allowed to exist in its present condition, having regard to the immense awakening in relation to hospital administration which has taken place all over the country during the last quarter of a century.

